# Editorial

**Published:** 1891-06

**Authors:** 


### Editorial.

### THE STATE MEDICAL ASSOCIATION.

The forty-second annual meeting of the Georgia State Medical Association was held in Augusta last month. This statement may prove a surprise to some, but nevertheless it is true. The minority who attended the meeting enjoyed it to much advantage, while the majority of the regular practicing physicians in Georgia took so little interest in this meeting that they did not attend it, or they staid away through sheer ig norance of its existence. The above statement is a bold yet lamentable fact. The membership of the Association is all that could be desired, as far as numbers are concerned, consisting, as it does, of all the prominent physicians in the State. And yet the meetings do not strike us as representing the whole Association in a unit. We scan the proceedings of the meetings for a number of years back, and we are struck with the constancy with which we see papers read by the same men, discussions conducted by a limited few, and motions made and seconded by those whose names ever and anon flit across our visual field. Surely, this should not be. Shall not the Georgia Medical Association represent the whole profession? There are scattered among the "red hills of Georgia," among its mountains and valleys, by the water's edge of that never restless sea, men of ability; men whose acquaintanceship would occasion both a pleasure and a profit to us, and yet many of them we never see.

By contact with others new ideas are evolved, the mind is sharpened and the wits are tested. It causes a man to think and reason upon the rapid advancements now being made to formulate and have ideas of his own, and thus check himself from treading upon an old and beaten path. Sims possessed those germs of knowledge which afterwards made him a pioneer and benefactor in his special line of work; yet it was his contact with men of more advanced ideas, absorbing those principles he thought good, and rejecting others, that caused him to rise to his height of distinction.

But illustrations are legion. We want the medical profession of Georgia to feel that each member of its Association is just as necessary for its welfare as any other, and let each one try to find out more about the Association to which he belongs. The first thing a young physician does after settling down to practice is to let some good brother physician present his name for membership in the State Medical Association, thus deceiving himself into the belief that he has done all that is requisite for a good standing in the medical profession. Delusive idea!

If ever a physician needs money, it is when he first begins to practice medicine, and money invested in the above way—simply the securing of a membership—is not "bread cast upon the waters." His money, so invested, had much better be used in the payment of office rent. We do not deprecate the idea of increased membership in the Association, but our plea is for greater unanimity of the *whole* membership—individual interest towards its success. Let all the members feel that when the annual meeting occurs that there will be a representation of the whole Georgia profession, and that acquaintanceships will be made and ideas expressed by representatives from every portion of the Empire State. There is not a member of the New York Academy of Medicine who does not esteem his membership with pleasure, and in every country there exists an association of some kind, to be recognized members of which is looked upon with pride by every physician. To be an active member, and one whose words are listened to with interest, is a still greater honor. Let it be that the Association of Georgia will be proud of the interest taken in it by its members. Let its meetings be a State representation, and let every physician feel that his ideas expressed will be as welcomely received as if he was the president of the Association.

---

The forty-second annual meeting of the American Medical Association was held in Washington on May 5th, 6th, 7th and 8th. Though we, ourselves, were unable to be present, the reports received and read of the meeting were of the most flattering character. A full account of which will be seen in this issue of the Journal. It is with pleasure that we note the election of Dr. J. M. Gaston, as Chairman of the Surgical Section for the ensuing term.

### A SUBJECT FOR THOUGHT.

Massachusetts and Georgia have been called the homes of quacks. While we cannot speak from experience of the condition of affairs which provides our New England neighbor, we are compelled to say that our own State seemingly warrants its appellation, judging from the laxness of her medical laws and the pride with which she nurtures the patent medicine fiends. Even the optician must delineate upon the pathological state of the public's visual organ and show them by plates, the necessity of a correcting glass. Surely we are returning to the mediaeval ages, judging from the credulity of many.

---

Through an oversight, the thanks due the New York Medical Abstract for the use of the cuts in the paper on "Cephalhaematoma and Teratomata," in our May issue were omitted.

---

TREATMENT OF FELONS.—D. B. D. Beavers, M. D., *Univerisity Med. Mag.*, April, 1891.

Advises early incision, being certain to strike the pus. He makes, in the first 24 or 48 hours after appearance of pain, a funnel-shaped incision with a straight bistoury, about half agian as heavy as a well made Graefe cataract knife.

To find location of pus point for incision, make pressure with end of probe, pressing gently over swollen part until sensitive pcint is found, which, on deeper pressure, the patient says "hurts to the bone," while around it deeper pressure is better borne. At this tender spot introduce the small bistoury down to bone, tilt handle, making a funnel-shaped opening at base, with little pain. Often a small drop of pus will follow withdrawal of knife, and patient be well in day or two. If left for three or four days or more to be certain of location and pus formation, serious damage is done—a long, free incision required, several days of suffering and slow recovery results.

